# Adult mouse and human organoids derived from thyroid follicular cells and modeling of Graves’ hyperthyroidism

**DOI:** 10.1073/pnas.2117017118

**Published:** 2021-12-16

**Authors:** Jelte van der Vaart, Lynn Bosmans, Stijn F. Sijbesma, Kèvin Knoops, Willine J. van de Wetering, Henny G. Otten, Harry Begthel, Inne H. M. Borel Rinkes, Jeroen Korving, Eef G. W. M. Lentjes, Carmen Lopez-Iglesias, Peter J. Peters, Hanneke M. van Santen, Menno R. Vriens, Hans Clevers

**Affiliations:** ^a^Hubrecht Institute, Royal Netherlands Academy of Arts and Sciences and University Medical Centre Utrecht, 3584 CT Utrecht, The Netherlands;; ^b^Oncode Institute, 3584 CT Utrecht, The Netherlands;; ^c^Maastricht Multimodal Molecular Imaging Institute, Maastricht University, 6229 ER Maastricht, The Netherlands;; ^d^Central Diagnostic Laboratory/Centre of Translational Immunology, University Medical Center (UMC) Utrecht, 3584 CX Utrecht, The Netherlands;; ^e^Department of Surgical Oncology, UMC Utrecht Cancer Center, 3584 CX Utrecht, The Netherlands;; ^f^Central Diagnostic Laboratory, University Medical Centre Utrecht, 3584 CX Utrecht, The Netherlands;; ^g^Princess Máxima Center for Paediatric Oncology, 3584 CS Utrecht, The Netherlands;; ^h^Department of Paediatric Endocrinology, Wilhelmina Children Hospital, University Medical Center Utrecht, 3584 EA Utrecht, The Netherlands

**Keywords:** thyroid, organoids, Graves’ disease

## Abstract

The thyroid maintains systemic homeostasis by regulating serum thyroid hormone concentrations. Here we report the establishment of three-dimensional (3D) organoids from adult thyroid tissue representing murine and human thyroid follicular cells (TFCs). The TFC organoids (TFCOs) harbor the complete machinery of hormone production as visualized by the presence of colloid in the lumen and by the presence of essential transporters and enzymes in the polarized epithelial cells that surround a central lumen. Both the established murine as human thyroid organoids express canonical thyroid markers PAX8 and NKX2.1, while the thyroid hormone precursor thyroglobulin is expressed at comparable levels to tissue. Single-cell RNA sequencing and transmission electron microscopy confirm that TFCOs phenocopy primary thyroid tissue. Thyroid hormones are readily detectable in conditioned medium of human TFCOs. We show clinically relevant responses (increased proliferation and hormone secretion) of human TFCOs toward a panel of Graves’ disease patient sera, demonstrating that organoids can model human autoimmune disease.

## Significance

The thyroid is essential for maintaining systemic homeostasis by regulating thyroid hormone concentrations in the bloodstream. This study describes an organoid-based model system to study mouse and human thyroid biology. Moreover, the study explores the potential of human organoids for modeling autoimmune disease, the anti-TSH receptor (TSHR) antibody-driven Graves’ hyperthyroidism.

The thyroid gland is composed of two histologically distinguishable cell types characterized by their representative hormone secretion: parafollicular cells, responsible for the secretion of calcitonin, and follicular cells, which secrete the main thyroid hormones (THs) triiodothyronine (T3) and thyroxine (T4). Parafollicular cells (also known as clear cells [C cells]) are relatively rare cells compared to the epithelial thyroid follicular cells (TFCs). TFCs line the follicles in which thyroid hormones are stored. This allows for a rapid release of thyroid hormones upon stimulation of the gland by thyroid stimulating hormone (TSH) secreted by the pituitary gland. A main component of TH, iodine, is transported from the circulation through TFCs into the follicle lumen. Transmembrane thyroid peroxidase (TPO) binds luminal iodine to tyrosine residues in the carrier protein thyroglobulin (TG). Upon stimulation by TSH, T3 and T4 coupled TG are reabsorbed into TFCs. Through lysosomal degradation, T3 and T4 are released from TG and secreted into the blood stream. Negative T3/T4-driven feedback loops block secretion of TSH, resulting in homeostasis (1). Both T3 and T4 affect many organ systems. The importance of maintaining thyroid hormone homeostasis is exemplified by the global incidence of thyroid-related diseases related to thyroid imbalance.

Graves’ disease is a thyroid-specific autoimmune disorder resulting in increased levels of thyroid hormones with subsequent low levels of TSH. The incidence of Graves’ hyperthyroidism in the Western world is around 20 cases per 100,000 individuals (2–4). Graves’ disease is characterized by the presence of antithyroid antibodies against the TSH receptor (TSHR), which activate downstream pathways, thus mimicking activation by TSH. This results in overproduction of T3 and T4 and subsequent low levels of TSH. Graves’ disease patients suffer from a variety of symptoms due to thyrotoxicosis, including an enlarged thyroid, anxiety, and tremor, and may have bulging eyes due to swelling of the tissue in the orbit (5). Current treatment consists of antithyroid therapy, reducing the levels of T3 and T4 and normalizing TSH levels (6). Approximately half of the patients treated with antithyroid drugs ultimately require additional treatment such as radioiodine or thyroidectomy, often resulting in hypothyroidism for which patients have to take lifelong hormone replacement (7). Generation of an in vitro model system that would allow for better understanding of thyroid-related diseases such as Graves’ disease could potentially lead to the development of alternative treatment options. Moreover, the generation of a transplantable thyroid culture system could potentially restore normal thyroid function.

Several different model systems have been employed to study thyroid biology and Graves’ disease. As early as in the 1950s, two-dimensional (2D) TFC layers were used as short-term thyroid cultures (8). These 2D layers, however, do not form normal physiological follicles and can’t be propagated. Submerging TFCs in suspension cultures, including extracellular matrix components, generates three-dimensionl (3D) polarized spheroids with a follicle architecture, but still cannot be maintained long term (9). Alternatively, mouse models have shown potential as model systems for thyroid biology and Graves’ disease modeling. The cost and time effectiveness of these studies, however, remains a limiting factor (10). These limitations can potentially be overcome by human adult stem cell–derived organoids. These three-dimensional cultures are representative in vitro model systems, which recapitulate human physiology (11). While we were finalizing the current study, Coppes and colleagues reported a first description of transplantable human (and mouse) thyroid organoids (12). In this study, we report similar findings and apply thyroid follicular cell organoids (TFCOs) to model Graves’ disease using patient sera.

## Results

**Fig. 1.**
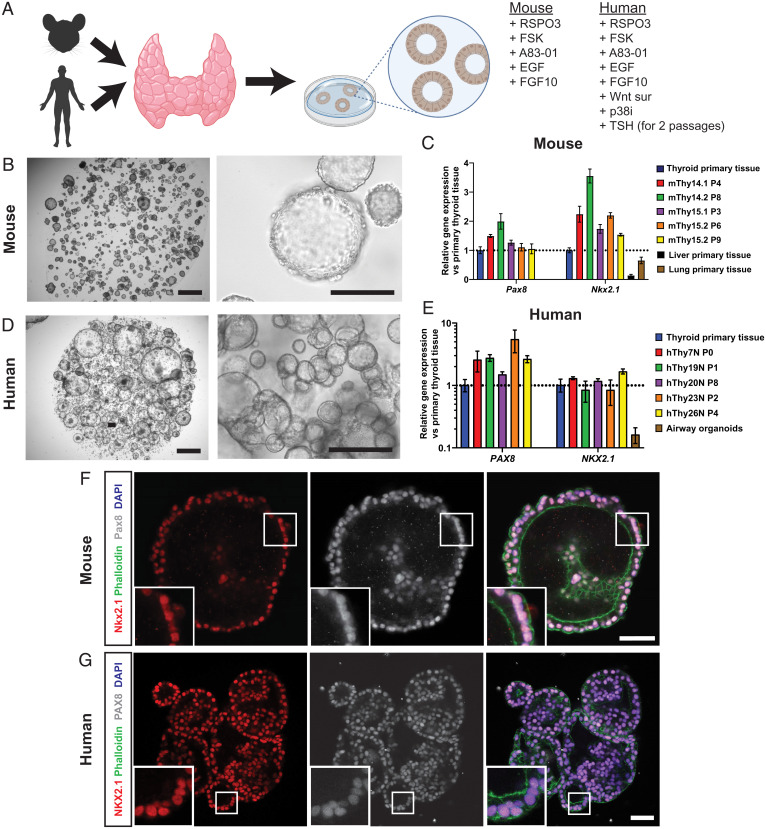
Establishment of thyroid follicular cell organoids from mouse and human origin. (*A*) Schematic representation of organoid derivation of TFCOs including medium components required for the establishment of murine TFCOs. Human TFCOs require additional components for establishment. (*B*) Brightfield images of murine TFCO cultures show cystic organoids grown in BME. (Scale bars, 1 mm [*Left*], 100 µm [*Right*].) (*C*) RT-qPCR analysis reveals similar to increased levels of Pax8 and Nkx2.1 expression in mouse TFCOs compared to primary thyroid tissue in early (fewer than four passages) and late (more than six passages) TFCOs while lung and liver tissue shows lower expression levels. *n* = 3. Error bars = SD. (*D*) Brightfield images of human TFCO cultures show cystic organoids grown in BME. (Scale bars, 1 mm [*Left*], 250 µm [*Right*].) (*E*) RT-qPCR analysis reveals similar to increased levels of PAX8 and NKX2.1 expression in human TFCOs compared to primary thyroid tissue in early (fewer than four passages) and late (more than six passages) TFCOs while lung tissue shows lower expression levels. *n* = 3. Error bars = SD. (*F* and *G*) Coexpression of thyroid transcription factors Nkx2.1/NKX2.1 (red) and Pax8/PAX8 (gray) in all cells in mouse (*F*) and human (*G*) TFCOs shows a pure population of TFCs in the organoids. Membrane staining was performed using Phalloidin (green), and nucleus staining using DAPI shows a single cell layer surrounding the lumen. (Scale bar, 100 µm.)

Based on our previously described 3D culturing conditions of long-term expansion of mouse and human organoids from a variety of other tissues, we dissected and minced thyroid tissue, incubated the fragments in digestion solution, and plated thyroid cells in basement membrane extract (BME). We optimized mouse TFCO medium by trial and error, to define a medium containing cyclic adenosine monophosphate (cAMP) activator forskolin (FSK), the Wnt potentiator R-spondin (RSPO), transforming growth factor beta (TGFβ) inhibitor A83-01, epidermal growth factor (EGF), and fibroblast growth factor 10 (FGF10). Human samples required the addition of Wnt surrogate (13), p38 inhibitor, and TSH before significant growth could be observed (Fig. 1*A*). Using these expansion media, organoids first formed after 4 d (mouse) or 1 to 2 wk (human) and continued to grow over a 3- to 6-wk period. Thereafter, organoids could be mechanically passaged at a ratio of 1:3 to 1:7 every 2 wk. The organoids could be maintained over 24 passages without significant change in morphology (to date we have reached passage 35 for mouse and passage 24 for human) (Fig. 1 *B* and *D*). The organoids were polarized in an “apical-in” orientation, as shown by the apical marker ZO-1 (*SI Appendix*, Fig. S1*A*). To confirm the cellular composition of the organoids, we used RT-qPCR, immunofluorescence, and immunohistochemistry. We observed expression of the generic TFC markers *Pax8/PAX8* and *Nkx2.1/NKX2.1* (formerly known as thyroid transcription factor-1) (TTF-1), in murine and human organoids. The expression appeared to be increased compared to primary thyroid tissue. Primary thyroid tissue, however, includes stromal cell types, which could explain this difference. The levels were comparable between different mouse and human donors (Fig. 1 *C* and *E*). By immunofluorescence, combinatorial nuclear expression of the two transcription factors (Nkx2.1/NKX2.1 and Pax8/PAX8) was confirmed in all cells in the organoids (Fig. 1 *F* and *G* and *SI Appendix*, Fig. S2 *A* and *B*). While both transcription factors are expressed in other tissues, including lung for *NKX2.1* and kidney for *PAX8*, coexpression of these transcription factors is unique to TFCs (14). Since all analyzed cells in the organoids were positive for both transcription factors, we concluded that the organoids mainly consisted of expanding populations of TFCs.

**Fig. 2.**
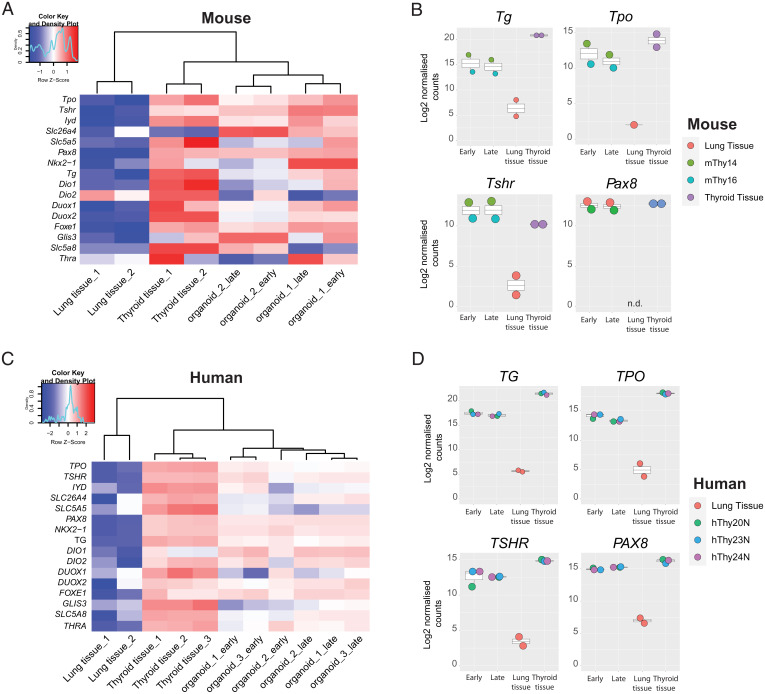
Bulk mRNA sequencing reveals limited differences between late and early passage TFCOs and primary thyroid tissue. (*A*) Heatmap indicating the expression of thyroid follicular cell markers in mouse primary thyroid tissue, lung tissue, and mouse TFCOs in early and late passages. TFCOs cluster with thyroid tissue in unsupervised hierarchical clustering and apart from lung tissue. Organoid 1 = mThy14, organoid 2 = mThy16. Colored bar represents row *z* scores of Log2 transformed normalized counts. Density plot indicates the fraction of genes with the given row *z* score. (*B*) Boxplots of mouse primary thyroid tissue (thyroid tissue), early passage TFCOs (early), and late passage TFCOs (late) as well as primary lung tissue (lung tissue) Log2 transformed normalized counts of bulk mRNA sequencing. Expression levels of Tg, Tpo, Tshr, and Pax8 are comparable to thyroid tissue. Color of the dots indicates donor visualized as described on the *Right*. Boxplot shows median, two hinges (25th and 75th percentile), and two whiskers (largest and smallest value no further than 1.5× interquartile range). n.d., not detected. (*C*) Heatmap indicating the expression of thyroid follicular cell markers in human primary thyroid tissue, lung tissue (GSE148815), and human TFCOs in early and late passages. TFCOs cluster with thyroid tissue in unsupervised hierarchical clustering and apart from lung tissue. Organoid 1 = hThy20N, organoid 2 = hThy23N, and organoid 3 = hThy24N. Colored bar represents row *z* scores of Log2 transformed normalized counts. Density plot indicates the fraction of genes with the given row *z* score. (*D*) Boxplots of human primary thyroid tissue (thyroid tissue), early passage TFCOs (early), and late passage TFCOs (late) as well as primary lung tissue of GSE148815 (lung tissue) Log2 transformed normalized counts of bulk mRNA sequencing. Expression levels of TG, TPO, TSHR, and PAX8 are comparable to thyroid tissue. Color of the dots indicates donor visualized as described on the *Right*. Boxplot shows median, two hinges (25th and 75th percentile), and two whiskers (largest and smallest value no further than 1.5× interquartile range).

To further characterize the TFCOs, we performed bulk mRNA sequencing on organoids at early and late passages for comparison with thyroid tissue and airway tissue (Datasets S1 and S2), the latter as unrelated control tissue. Hierarchical clustering of the samples based on known thyroid-expressed genes showed extensive overlap between organoid samples and thyroid tissue samples, while clustering away from the lung tissue (Fig. 2 *A* and *C*). Both murine and human TFCO samples showed comparable levels of, i.e., *TPO*, *PAX8*, and *DUOX2* to primary tissue. TFCOs were stable as deduced from early and late passage TFCO expression data. When single genes were extracted and compared to primary tissue data, we observed a slight decrease in levels of some thyroid maturation markers like *TG* in both mouse and human TFCOs (Fig. 2 *B* and *D*). This could be due to the proliferative nature of cells within organoids compared to the in vivo situation. This proliferative nature is confirmed by the expression of MKI67 in a subset of cells within the organoids while being absent in the thyroid tissue (*SI Appendix*, Figs. S1*B*/S2*C*). Thyroid stem-like cells have been discussed for decades without a general consensus (15, 16); the proliferative cells in the TFCOs likely represent their in vitro counterparts. We analyzed the presence of proposed stem cell markers in human TFCOs. Since we observed a gradually increasing growth rate from passage 5 onwards, we hypothesized that such stem cell markers would become evident when comparing tissue to early passage TFCOs and would increase in expression from early to late passage organoids. No significant change in expression was identified in the putative stem cell markers *SCA1* (17, 18), *ABCG2* (17, 19), *FUT4* (12), *GATA4* (20), *HNF4A* (20), *NANOG* (21), *OCT4* (12, 17, 20–22), and *HHEX* (15, 23) in human TFCOs (*SI Appendix*, Fig. S3 *A*–*J*). Increased expression was noted for NGFR and FOXE1 (TTF2) (24), while SOX2 (12, 22) was enriched in late passage human TFCOs (*SI Appendix*, Fig. S3 *K*–*M*). Of note, the latter three genes were not up-regulated in mouse TFCOs compared to tissue (*SI Appendix*, Fig. S3 *N*–*P*).

**Fig. 3.**
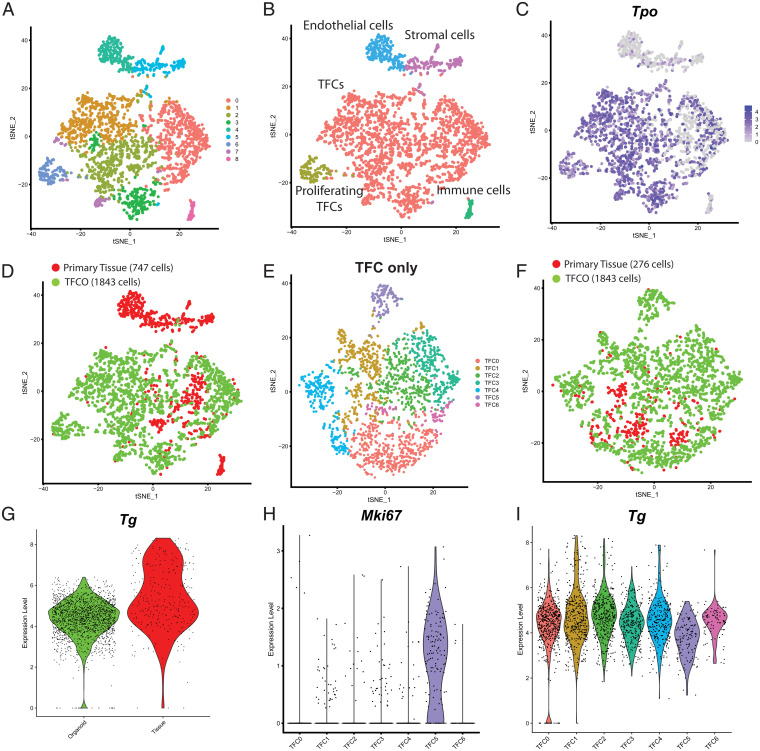
Single-cell RNA sequencing of mouse TFCOs and primary tissue reveals limited differences between TFCs except for a proliferative population in organoids. (*A*) t-distributed stochastic neighbor embedding (tSNE) representation of nine clusters of mouse thyroid tissue cells (*n* = 747) and mouse TFCO cells (*n* = 1,843). (*B*) tSNE representation of five cell types of mouse thyroid tissue cells (*n* = 747) and mouse TFCO cells (*n* = 1,843). (*C*) tSNE representation of the expression of TFC-specific peroxidase Tpo (linear scale). (*D*) tSNE representation of two origins of mouse thyroid tissue cells (*n* = 747) and mouse TFCO cells (*n* = 1,843) visualizing overlap between tissue-derived and TFCO-derived TFCs. (*E*) tSNE representation of seven clusters of mouse thyroid tissue TFCs (*n* = 276) and mouse TFCO TFCs (*n* = 1,843) after reclustering. (*F*) tSNE representation of two origins of mouse thyroid tissue TFCs (*n* = 276) and mouse TFCO TFCs (*n* = 1,843) after reclustering, visualizing the overlap between tissue-derived and TFCO-derived TFCs. (*G*) Violin plots of the normalized expression level of Tg in TFCOs (“organoid,” *n* = 1,843 cells) and primary thyroid tissue (“primary tissue,” *n* = 276 cells) show similarity in expression levels with the exception of some more mature TFCs in thyroid tissue. (*H*) Violin plots of the normalized expression level of Mki67 in TFC clusters indicates the presence of a proliferative subpopulation of TFCs in cluster TFC5. (*I*) Violin plots of the normalized expression level of Tg in TFC clusters indicates a less mature population of TFCs in cluster TFC5.

To further delve into cellular heterogeneity in the organoids, we performed single-cell sequencing (25) on mouse primary tissue and early and late passage mouse TFCOs. We sorted and processed, using the SORT-seq method (26), a total of 747 primary tissue cells and 1,843 TFCO cells which passed quality control. Clustering of the cells revealed nine clusters which could be annotated using known marker genes (Fig. 3*A*). Some clusters were only composed of cells originating from primary tissue, including 58 monocytes expressing *Ptprc* and *Lyz2*, 197 stromal cells expressing *Col3a1* and 216 endothelial cells expressing *Ly6a* (Fig. 3*B* and *SI Appendix*, Fig. S4). Some *Calca*-expressing cells could be identified within the tissue cell sample, indicative of parafollicular/C cells. A single Calca expressing cell was identified in the organoids underscoring the rapid loss of these neural crest-derived cells in the epithelial TFCOs (*SI Appendix*, Fig. S4 *G* and *H*). This observation was confirmed by the loss of Calca expression as determined by RT-qPCR (*SI Appendix*, Fig. S4*I*). The majority of the cells (2,119 cells, 276 cells from primary tissue and 1,843 cells from TCFOs) expressed *Pax8*, *Tpo*, and *Nkx2.1* and could therefore be annotated as TFCs (Fig. 3 *B* and *C*). Satisfyingly, TFCs from the primary tissue clustered with TFCs from TFCOs underscoring the high similarity between cultured and primary TFCs (Fig. 3*D*). After reclustering of all TFCs, seven TFC clusters became evident (Fig. 3*E*), while tissue TFCs continued to cocluster with TFCO TFCs (Fig. 3*F*). *Tg* levels between TFCs from organoids and primary tissue cells were similar, yet rare cells with high *Tg* levels were only seen in primary tissue (Fig. 3*G*).

Differential expression analysis between the clusters revealed relatively minor differences between the clusters with the exception of cluster 5, which uniquely expressed multiple genes involved in cell cycling, including *Mki67*, *Ccnb2*, and *Pcna* (Fig. 3*H*). In agreement, cluster 5 contained only organoid-derived TFCs (Fig. 3 *E* and *F*) while expression levels of the TFC maturation marker Tg was slightly lower compared to the other clusters (Fig. 3*I*). Thus, cluster 5 cells represented the proliferative TFC progenitors, also seen by immunofluorescence (*SI Appendix*, Fig. S1*B* and S2*C*). The relative proportion of proliferative cells (within cluster 5 of each line) ranged between 4 and 13%. Since this subpopulation could potentially contain the elusive thyroid stem cells, we performed differential expression analysis and identified 148 significantly (*P* < 0.01) enriched genes (Dataset S3). None of the proposed stem cell markers (see above) were present in this list or were enriched in the proliferative TFC cluster with the exception of *Hhex*. *Hhex* was enriched in proliferative TFCs compared to other TFCs (*SI Appendix*, Fig. S5 *A* and *B*) as well as in late passage TFCOs compared to early passage TFCOs and primary tissue (*SI Appendix*, Fig. S5*C*). Mouse *Hhex* expression was observed by bulk mRNA-sequencing data to be higher in TFCO cultures than in primary tissue (*SI Appendix*, Fig. S5*D*) while *HHEX* levels in human TFCOs were similar to primary tissue (*SI Appendix*, Fig. S3*J*). It was therefore unlikely that HHEX performs similar functions in human and mouse TFCs. The lack of a single population and the high comparability between the populations might indicate that thyroid tissue and organoids do not harbor professional stem cells. The growth of organoids may therefore be due to the presence of a transient proliferative cell pool with high resemblance to mature TFCs. Moreover, we concluded that the single-cell atlas of the mouse thyroid and TFCOs showed high similarity between TFCs derived from primary tissue and from TFCOs as well as within the TFC population.

**Fig. 4.**
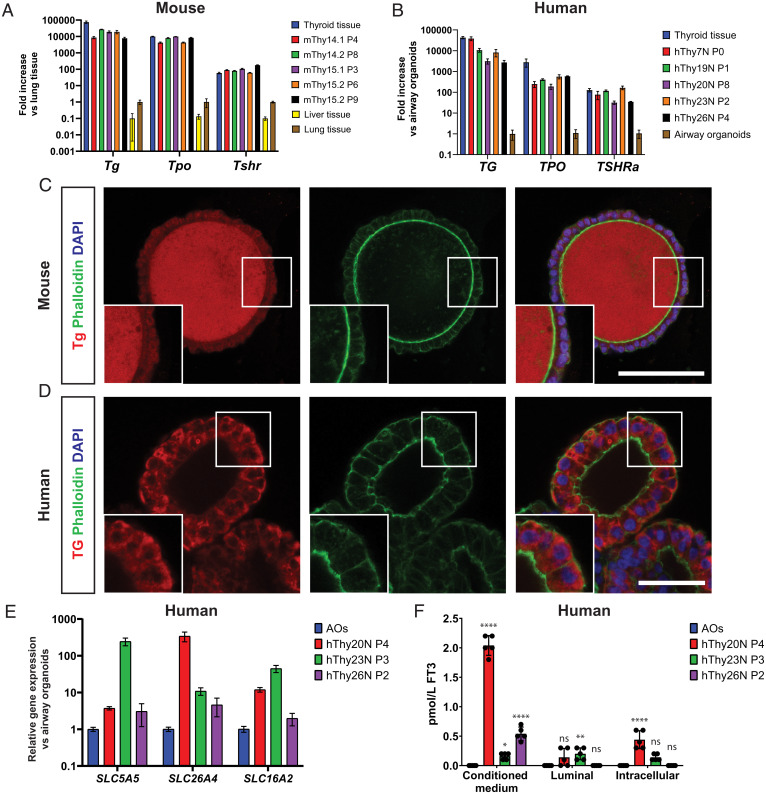
TFCOs express thyroid hormone machinery and secrete thyroid hormone basally. (*A*) RT-qPCR analysis reveals similar levels of Tg, Tpo, and Tshr expression in mouse TFCOs compared to primary thyroid tissue in early (fewer than four passages) and late (more than six passages) TFCOs, while liver tissue shows lower expression levels. Expression levels are relative to lung tissue expression levels. *n* = 3. Error bars = SD. (*B*) RT-qPCR analysis reveals slightly lower yet significant levels of TG and TPO and similar levels of TSHRa expression in human TFCOs compared to primary thyroid tissue in early (fewer than four passages) and late (more than six passages) TFCOs. Expression levels are relative to airway organoid expression levels. *n* = 3. Error bars = SD. (*C*) Mouse TFCs secrete Tg (red) toward the lumen in TFCOs similar to TFCs in follicles in vivo. Membrane staining was peformed using Phalloidin (green) and nucleus staining using DAPI (blue). (Scale bar, 100 µm.) (*D*) Human TFCs express TG (red) cytoplasmically in TFCOs. Membrane staining was performed using Phalloidin (green) and nucleus staining using DAPI (blue). (Scale bar, 50 µm.) (*E*) RT-qPCR analysis reveals expression of iodine transporters SLC5A5 and SLC26A4 and thyroid hormone transporter SLC16A2 in human TFCOs. Expression levels are relative to airway organoid (AO) expression levels. *n* = 3. Error bars = SD. (*F*) Human TFCOs in varying passages secrete variable yet measurable levels of free T3 (FT_3_) basally (conditioned medium) while apical secretion (luminal) is minimal or nondetectable. Some levels of FT_3_ are identified intracellularly (intracellular) yet at lower levels compared to basally. Each dot represents a separate expanded well of organoids measured. *n* = 5. Error bars = SD. ns, not significant, *P < 0.05, **P < 0.01, ****P < 0.0001 using two-way ANOVA with Tukey’s multiple comparisons to AOs.

Since expression levels indicated maturity and functionality of the TFCOs, we examined the presence of the thyroid hormone machinery in murine and human TFCOs (Fig. 4). In agreement with the sequencing data, TFCOs of murine and human origin both expressed high levels of *Tg/TG* and *Tpo/TPO*, essential for generation and storage of thyroid hormones (Fig. 4 *A* and *B*). Expression of *Tshr/TSHR* was similar between primary tissue and TFCOs from both species (Fig. 4 *A* and *B*). This indicated the potential of TFCOs to respond to TSH. TG is the carrier protein of thyroid hormones and allows for rapid hormone secretion without the need for transcription and translation; it is stored as colloid in the lumen of follicles. Five days after splitting, murine TFCOs contained a population of Tg-expressing cells (*SI Appendix*, Fig. S1*C*). When these TFCOs were maintained for 21 d without splitting, the lumens were filled with Tg, resembling the in vivo situation (Fig. 4*C* and *SI Appendix*, Fig. S2*D*). Human TFCOs expressed cytoplasmic Tg in most if not all cells, while luminal TG was much less prominent (Fig. 4*D*). This was likely due to the repeated swelling and bursting of human TFCOs (not observed in mouse TFCOs), potentially leading to continuous loss of luminal Tg. To document the potential of iodine transport and thyroid hormone production by human TFCOs, we analyzed the expression of iodine transporters *SLC5A5* (sodium/iodine symporter) (NIS) and *SLC26A4* (Pendrin) as well as the T3 transporter *SLC16A2* (MCT8). All three transporters were expressed in human TFCOs (Fig. 4*E*). Combined with the expression of *TPO* and *TG*, this indicated functionality of hormone production and secretion of human TFCOs. Secretion of thyroid hormones in the form of free T3 (FT_3_) was successfully observed in medium collected from TFCOs 24 h after replacing the medium by phosphate-buffered saline (PBS) (Fig. 4*F*). Thus, TFCOs possessed the thyroid hormone machinery and could secrete thyroid hormones from their basal side.

**Fig. 5.**
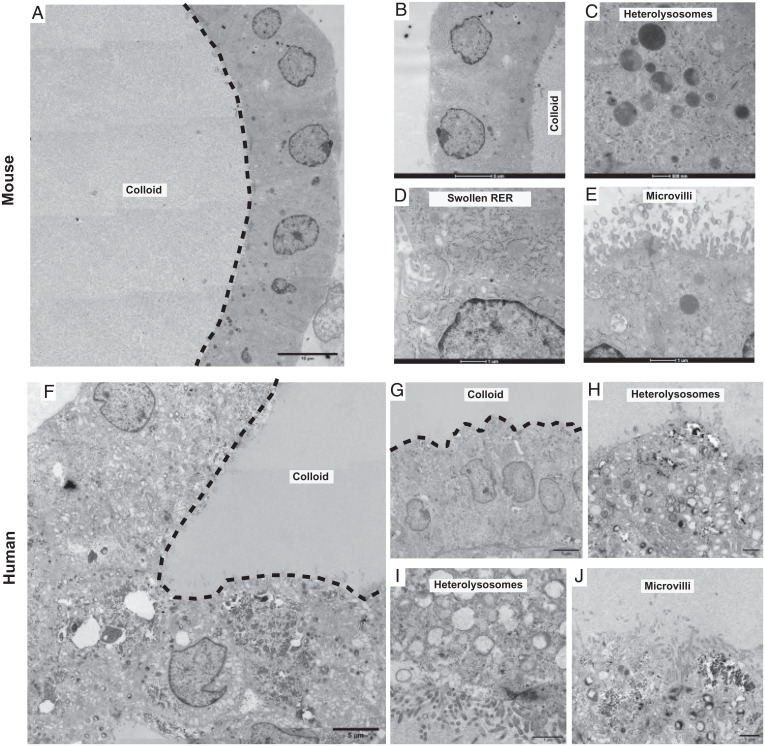
TFCs show short microvilli, swollen RER cisternae, and heterolysosomes and electrondense follicular lumens in TFCOs. (*A*) Overview image of transmission electron microscopy image reveals a variable in density lumen like the thyroid follicles show filled with potential colloid (dashed line indicates boundary of lumen) in mouse TFCOs. (Scale bar, 10 µm.) (*B*) Detailed zoom-in reveals a single cell layer of TFCs with cubic to columnar cells containing the nucleus in the basal area in mouse TFCOs. (Scale bar, 5 µm.) (*C*) Extensive heterolysosomes can be observed in mouse TFCOs indicating active transport of thyroglobulin to and from the lumen. (Scale bar, 500 nm.) (*D*) Swollen rough endoplasmic reticulum (RER) is present in mouse TFCOs indicative of TFCs. (Scale bar, 1 µm.) (*E*) Finger-like extensions of the plasma membrane can be observed as microvilli on the apical membrane of TFCs in mouse TFCOs. (Scale bar, 1 µm.) (*F*) Overview image of transmission electron microscopy reveals a variable in density lumen like the thyroid follicles show filled with potential colloid (dashed line indicates boundary of lumen) in human TFCOs. (Scale bar, 5 µm.) (*G*) Detailed zoom-in reveals a single cell layer with cubic to columnar cells containing the nucleus in the basal area of TFCs in human TFCOs. (Scale bar, 5 µm.) (*H* and *I*) Extensive heterolysosomes can be observed in human TFCOs, indicating active transport of thyroglobulin to and from the lumen. (Scale bars, 1 µm.) (*J*) Finger-like extensions of the plasma membrane can be observed as microvilli on the apical membrane of TFCs in human TFCOs. (Scale bar, 1 µm.)

We next performed transmission electron microscopy (TEM) on both mouse and human TFCOs. Only follicular cells were identified in a typical thyroid gland organization as a single layer of cells around the lumen. Colloid was identified in the lumen of most mouse and human TFCOs (Fig. 5 *A*, *B*, *F*, and *G*). Moreover, abundant potential small secretory and endocytic vesicles transporting thyroglobulin to and from the lumen and hetero- or endolysosomal vesicles created by the fusion of lysosomes with the vesicles reabsorbing thyroglobulin could be detected (Fig. 5 *C*, *H*, and *I*), indicative of active transport of Tg/TG and thyroid hormones. Underlining the active translation of carrier protein Tg and other thyroid hormones machinery, we observed typical swollen rough endoplasmic reticulum present in the follicular cells of the thyroid where thyroglobulin synthesis takes place (Fig. 5*D*). Additionally, short microvilli were observed on the apical membrane projecting into the colloid of mouse and human TFCs (Fig. 5 *E* and *J*) and the follicle cell junctional complexes restricting colloid to the lumen (Fig. 5*E*). All these features agree with earlier reports of transmission electron microscopic studies of rat thyroid (27–29).

**Fig. 6.**
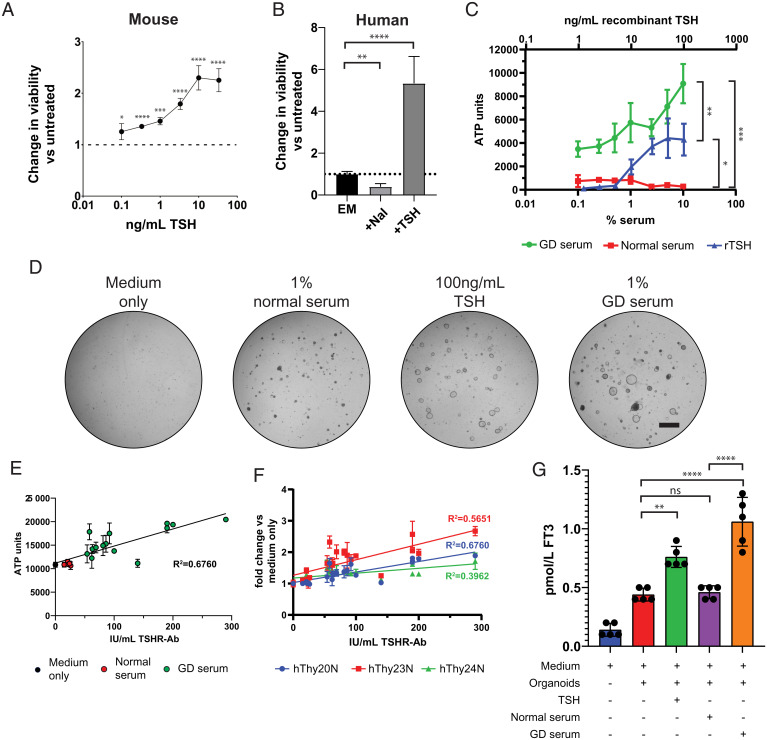
TFCOs model Graves’ disease by increased proliferation and hormone secretion after TSHR-Ab stimulation. (*A*) Addition of TSH increases viability of mouse TFCOs compared to TFCOs cultured without TSH after 7 d. All data points are relative to TFCOs grown in the absence of TSH and FSK. *n* = 3 per data point. Error bars = SD. *P < 0.05, ***P < 0.001, ****P < 0.0001 using two-way ANOVA with Tukey’s multiple comparisons to untreated samples. (*B*) Human TFCOs increase viability upon addition of 100 ng/mL TSH (+TSH) as compared to expansion medium (EM) after 7 d. Addition of sodium iodide (NaI) decreases viability of human TFCOs as compared to EM. *n* = 3. Error bars = SD. **P < 0.01, ****P < 0.0001 using two-way ANOVA with Tukey’s multiple comparisons. (*C*) Increased viability is observed in human TFCOs when cultured for 10 d with 10 to 100 ng/mL recombinant (rTSH) (*Upper* axis) compared to untreated TFCOs. More extensive increase in viability is observed when TFCOs are cultured for 10 d with serum of Graves’ disease patients (TSHR-Ab >3.3 IU/L) (*Lower* axis). Incubation with normal serum (TSHR-Ab <3.3 IU/L) did not show increased viability of TFCOs even when using 10% serum (*Lower* axis). *n* = 3. Error bars = SD. *P < 0.05, **P < 0.01, ***P < 0.001 using two-way ANOVA with Tukey’s multiple comparisons. (*D*) Representative brightfield images show limited outgrowth in expansion medium without FSK (medium only) and 1% normal serum (TSHR-Ab <3.3 IU/L) after 10 d. Increased outgrowth is visible in cultures grown with 100 ng/mL TSH after 10 d. More extensive outgrowth and organoid size is observed in cultures grown in 1% GD serum (TSHR-Ab >3.3 IU/L). (Scale bar, 1 mm.) (*E*) Incubation of TFCOs with 1% serum with varying TSHR-Ab titers reveals correlation between TFCO growth and TSHR-Ab titers. Each dot represents a single serum tested in triplicate. Color of the dots indicate category of serum being medium only (black), normal serum (TSHR-Ab <3.3 IU/L) (red), or GD serum (TSHR-Ab >3.3 IU/L) (green). *n* = 3 per data point. Error bars = SD. R2 using Pearson correlation. (*F*) Comparison between three different donors (indicated in different colors) in a similar assay as in *E* shows correlation of TSHR-Ab titers and TFCO growth in all donors still with variable slopes. *n* = 3 per data point. Error bars = SD. R2 using Pearson correlation. (*G*) Stimulation with 100 ng/mL recombinant TSH or 1% GD serum (TSHR-Ab >3.3 IU/L) increases free T3 (FT_3_) levels in conditioned medium of human TFCOs after 48 h. No significant increase in FT_3_ levels is observed when TFCOs are stimulated with 1% normal serum (TSHR-Ab <3.3 IU/L) compared to medium in combination with organoids. Each dot represents a separate expanded well of organoids measured. *n* = 5. Error bars = SD. ns, not significant, **P < 0.01, ****P < 0.0001 using two-way ANOVA with Tukey’s multiple comparisons.

TSH stimulates the expression of iodine transporter *SLC5A5*, stimulates hormone formation and TG endocytosis and proteolysis, and induces the secretion of thyroid hormones. Prolonged TSH stimulation of the thyroid gland induces hyperplasia (30, 31). Given the expression at physiological levels of TSHR, which signals through cAMP (32, 33), we performed growth assays upon addition of TSH. Adenosine triphosphate (ATP) levels in the CellTiter Glo assay were used as surrogate for proliferation. Since Forskolin (FSK) also activates cAMP signaling, we removed FSK from the medium. Satisfyingly, growth of mouse TFCOs as well as human TFCOs was increased when assayed over a period of 7 d upon TSH stimulation (Fig. 6 *A* and *B*) in a dose-dependent manner (Fig. 6*A*). Contrarily, we observed reduced growth upon addition of iodine (sodium iodide) (NaI) in human TFCOs (Fig. 6*B*). In patients with Graves’ disease, the anti-TSHR autoantibodies overactivate the TSH pathway, resulting into hyperthyroidism. As shown above, TFCOs respond to TSH. To test whether the TFCOs would also respond to TSHR-Abs, we incubated human TFCOs with sera from a Graves’ disease patient (GD serum) (TSHR-Ab titers >3.3 IU/L) at different dilutions for 10 d. The human TFCOs incubated with Graves’ disease serum showed increased growth compared to TFCOs incubated with control sera (TSHR-Ab titers <3.3 IU/L) (Fig. 6*C*). In agreement, some expansion of TFCOs was observed in wells incubated with 1% control serum while extensive outgrowth of TFCO was observed in wells incubated with 100 ng/mL TSH or 1% Graves’ disease sera (Fig. 6*D*).

To further validate the use of TFCOs as in vitro models of Graves’ disease, we tested 15 Graves’ disease sera and 5 control sera in parallel at a 1% dilution and compared ATP units using the CellTiter Glo assay (*Materials and Methods*). We observed a positive correlation between the anti-TSHR antibody titers and ATP levels in TFCOs (*R*^2^ = 0.6760) (Fig. 6*E*). Two independent donors showed similar correlations (*R*^2^ = 0.5651 and 0.3962) underlining the robustness of TFCO culture (Fig. 6*F*). Lastly, we measured FT_3_ levels in organoids after stimulation with TSH, a selected potent Graves’ disease serum and control serum. TFCOs yielded background levels of 0.45 pmol/L FT_3_ in the conditioned medium after an incubation of 48 h. Incubation with 100 ng/mL TSH or 1% Graves’ disease serum increased FT_3_ levels to 0.75 and 1.05 pmol/L respectively, while TFCOs incubated with 1% normal serum yielded similar levels of FT_3_ (Fig. 6*G*). In conclusion, TFCOs recapitulate characteristics of Graves’ disease (growth and hormone release) when treated with Graves’ disease serum.

## Discussion

In this study, we describe the generation of adult thyroid follicular cell organoids from mouse and human origin. The establishment of these TFCOs requires limited starting material and the resulting organoid lines can be stably expanded for >1 y. The optimized protocol allowed the generation of 10 human organoid lines from 12 provided donor tissue samples (±83%). Several other groups have recently reported similar thyroid organoids derived from mouse (34), rat (35), and human tissue (12). Related approaches have resulted in the establishment of human thyroid cancer organoids (36, 37). Our TCFO lines faithfully recapitulate TFC function and TSH responses. Our human TFCOs secrete thyroid hormone under expansion conditions, which contrasts with the previously published human thyroid organoids where transfer to a maturation medium was required (12). Current expansion conditions showed stable expression of essential thyroid-related genes, even after >1 y of culture, while loss of thyroid differentiation was observed in organoids generated by Ogundipe et al. over time in culture (12). These differences may result from differences in medium composition, yet we conclude that both studies provide definitive evidence that thyroid organoids can be established and that these faithfully recapitulate the primary tissue.

Using single-cell mRNA sequencing, a proliferative population could be identified, but unlike the original Lgr5^+^ stem cell–driven gut organoids (38), we found no evidence for the existence of unique thyroid stem cells in our in vitro model system. It has emerged recently that many solid tissues don’t rely on a “professional” stem cell for their maintenance and repair, but rather temporarily recruit differentiated cells into a transient progenitor pool (39). The liver is likely the most explicit example of this phenomenon, where—depending on the type of damage—either fully differentiated cholangiocytes or hepatocytes transiently acquire proliferating progenitor phenotypes to regenerate lost tissue (40). We conclude that our current observations suggest that a similar phenomenon may play out in the thyroid. While adult thyroid turnover occurs on the order of 10 y, there are reports of thyroid regeneration after injury even though the gland does not tend to recover to its original size (18, 41–44).

TFCOs could become a potential source of thyroid transplantation. Currently, radioiodine treatment or thyroidectomy are required to treat hyperthyroidism or thyroid carcinoma, resulting in hypothyroidism followed by lifelong administration of thyroid hormones (45). Although thyroid hormone supplemental therapy can in theory “easily” be done by daily levothyroxine prescription, many patients complain of not being in optimal thyroid hormone balance. Autologous transplantation of TSH-responsive cultured TFCOs may in the future replace these external regulations and restore a physiological thyroid hormone balance with TSH-responsive thyroid tissue. Indeed, Coppes and colleagues have shown that their organoids (which are very similar to the ones described in the current study) can be transplanted to mice (12).

The requirement of TSH during the initial passages and FSK in further expansion of human TFCOs shows the dependence of TFC cells on the cAMP pathway. Activation of TSHR and thereby of the cAMP pathway by TSH has been shown to induce proliferation in TFCs (30, 31). This activation is mimicked by TSHR binding antibodies (5). These phenomena are readily recapitulated in our TFCOs. Recently, salivary gland organoids were generated from patients suffering from another autoimmune disorder, Sjögren’s syndrome, and underlying biological mechanisms of salivary hypofunction were identified (46). Together, this indicates the promise of organoids as models for autoimmune disorders. Since organoids have been shown to predict patient responses for cystic fibrosis and cancer (47–50), TFCOs can potentially aid the development of new or repurposed drugs for Graves’ disease.

In conclusion, we present adult mouse and human thyroid follicular cell organoids containing two populations of TFCs which are highly similar except for proliferation status. These TFCs recapitulate essential characteristics of the primary tissue and are responsive to external stimuli such as TSH and anti-TSHR antibodies.

## Materials and Methods

### Patient Samples.

Human thyroid biopsies were anonymously obtained from the Department of Surgery at the University Medical Center Utrecht (UMCU) from waste material of patients undergoing surgery at the UMCU. The use of samples for research and informed consent procedures were approved by the medical ethical committee (TCBio) of the UMCU as protocol 12-093 and was in accordance with the Declaration of Helsinki and according to Dutch law. This study is compliant with all relevant ethical regulations regarding research involving human participants. Informed consent was obtained prior to surgery. Samples were deidentified for the researcher to keep patient data at the medical center.

### Mice.

All animal experiments were performed after institutional review by the Animal Ethics Committee of the Royal Netherlands Academy of Arts and Sciences (KNAW) with project license AVD8010020151. Surplus material from female C57BL/6 mice was used to derive organoids in this study.

### Thyroid Organoid Culture.

Organoid derivation and analysis is adapted from earlier described protocols (51).

Human samples were kept in advanced (Ad) Dulbecco's Modified Eagle Medium (DMEM)/ F12 solution (GIBCO) with penicillin/streptomycin (Thermo Fisher Scientific) at 4 °C until further processing. For both mouse and human, complete or part of the gland was chopped into small ∼1-mm pieces using a scalpel. Tissue pieces were enzymatically digested in digestion medium for about 25 to 30 min of shaking (120 rpm) at 37 °C. Composition of the medium is further described in *SI Appendix*. The homogeneous cell suspension was pelleted and washed with AdDMEM/F12. The tissue was resuspended in 1 mL red blood cell lysis buffer (Roche, 11814389001) and incubated at room temperature for 5 min. Fragments were mechanically dissociated using narrowed glass pipettes. Cells from a single murine thyroid were plated in ∼100 µL Cultrex Pathclear Reduced Growth Factor BME (3533-001, Amsbio). For human biopsies the volume of BME was determined based on the size of the final pellet. After BME solidification, complete expansion medium was added. Splitting procedures are further described in *SI Appendix*.

### Viability Assays.

Cell viability was determined using CellTiterGlo 3D (Promega) according to the manufacturer’s instructions. More detailed protocols are discussed in *SI Appendix*.

### Immunohistochemistry and Imaging.

Organoids were harvested in Cell Recovery solution (354253, Corning) and fixed in 4% paraformaldehyde (Sigma-Aldrich) for at least 2 h at room temperature. Thyroid tissue used for immunohistochemistry was directly fixed in 4% paraformaldehyde upon dissection and incubated for at least 2 h at room temperature or overnight at 4 °C. Samples were washed and dehydrated by an increasing ethanol gradient and washed in xylene before embedding in paraffin. Sections were cut and hydrated before staining. Slides were imaged using a Leica DM4000 microscope.

For immunofluorescence, organoids were harvested, fixed, and permeabilized using 0.2% Triton X-100. Whole-mount staining was performed overnight in 2% goat serum. Organoids were imaged on a Leica SP8X or SP8 microscope.

Antibodies used in this manuscript are listed in *SI Appendix*.

### qPCR Analysis.

RNA isolations of organoids and tissue for bulk RNA sequencing and qPCR were performed with RNeasy Mini Kit (Qiagen) following the manufacturer’s instructions. Quantitative PCR analysis was performed using the SYBR Green and Bio-Rad systems. Changes in expression relative to housekeeping gene and thyroid or lung tissue when indicated were calculated using CFX Manager software (Bio-Rad). Primers were designed using the National Center for Biotechnology Information (NCBI) primer design tool and are indicated in *SI Appendix*.

### Bulk mRNA Sequencing.

Bulk mRNA sequencing was performed by the Utrecht Sequencing Facility (USEQ) using the TruSeq stranded mRNA kit (Illumina). In short, polyA-enriched RNA was reverse transcribed and paired-end reads were mapped to the mouse genome. Expression data were analyzed using DESeq2 (52).

### Single-Cell mRNA Sequencing.

For single-cell sequencing of the tissue, mouse thyroid was dissociated with collagenase I (Sigma-Aldrich) as described above and subsequently resuspended in TrypLE Express (GIBCO) preheated to 37 °C and dissociated under repeated pipetting. For single-cell sequencing of TFCOs, organoid droplets were incubated in Cell Recovery solution (354253, Corning) for 30 min in order to dissolve the BME. Then, organoids were pelleted and resuspended in TrypLE Express (GIBCO) preheated to 37 °C and dissociated under repeated pipetting. When the gland and the organoids were fully dissociated into single cells, samples were pelleted, washed, resuspended in fluorescence-activated cell sorting (FACS) buffer (advanced DMEM/F12, 10 μM Y-27632, and DAPI) and strained (35 μm).

DAPI-negative cells were immediately sorted into 384-well plates containing External RNA Controls Consortium (ERCC) spike-ins (Agilent), reverse transcription (RT) primers, and deoxynucleotide triphosphates (dNTPs) (Promega) using a FACSFusion (BD Biosciences). Plates were prepared using Mosquito HTS (TTP Labtech). Single-cell RNA-sequencing libraries were prepared following the SORT-seq protocol (26), which is based on the CEL-seq2 method (53). Extensive explanation of the protocol can be found in *SI Appendix*.

### Transmission Electron Microscopy.

Organoids were chemically fixed for 3 h at room temperature with 1.5% glutaraldehyde in 0.067 M cacodylate buffered to pH 7.4 and 1% sucrose. Samples were washed once with 0.1 M cacodylate (pH 7.4), 1% sucrose, and three times with 0.1 M cacodylate (pH 7.4), followed by incubation in 1% osmium tetroxide and 1.5% K_4_Fe(CN)_6_ in 0.1 M sodium cacodylate (pH 7.4) for 1 h at 4 °C. After rinsing with MilliQ (MQ), organoids were dehydrated at room temperature in a graded ethanol series (70, 90, up to 100%) and embedded in epon. Epon was polymerized for 48 h at 60 °C. The 60-nm Ultrathin sections were cut using a diamond knife (Diatome) on a Leica UC7 μLtramicrotome and transferred onto 50-mesh copper grids covered with a formvar and carbon film. Sections were poststained with uranyl acetate and lead citrate.

All TEM data were collected autonomously as virtual nanoscopy slides (54) on FEI Tecnai T12 microscopes at 120 kV using an Eagle camera. Data were stitched, uploaded, shared, and annotated using Omero (55) and PathViewer.

### Autoimmune Thyroid Disease Patient Serum.

Patient serua were anonymously obtained from Henny Otten at the UMCU from waste material of patients diagnosed at the UMCU. The use of sample for research was approved by the medical ethical committee (TCBio) of the UMCU as protocol 21-013 and was in accordance with the Declaration of Helsinki and according to Dutch law. This study is compliant with all relevant ethical regulations regarding research involving human participants.

TSHR autoantibodies were detected and quantified with the ELiA anti-TSHR assay with sample processing by the Phadia 250 instrument, performed as specified by the manufacturer (Thermo Fisher Scientific) as described before (56). Serum was categorized as Graves’ disease serum when TSHR-Ab titers were >3.3 IU/L and as normal serum when <3.3IU/L. Serum was aliquoted and stored at −20 °C until use.

### Free T3 Analysis.

Organoids were expanded as mentioned above. After organoids reached cystic morphology (4 to 6 d after splitting), medium was changed to experimental medium, which included indicated supplements. Medium was harvested after 48 h and kept at 4 °C for a maximum of 2 d or −20 °C for extensive periods until analysis. For luminal content, medium was removed and organoids were spun down and resuspended in 800 µL expansion medium. The organoids were broken using a narrowed glass pipette and spun down. Supernatant was collected and sent for analysis. For intracellular content, organoids were harvested, broken using a narrowed glass pipette, and sonicated for 15 cycles for 30 s and a 30-s pause using a Branson sonicator SFX250. Samples were spun down and supernatant was sent for analysis.

Analysis was performed by the Central Diagnostic Laboratory at UMCU. The FT_3_ measurements were performed, as specified by the manufacturer, on the Atellica IM analyzer (Siemens Healthineers). The lowest concentration that can be measured with 95% confidence is 0.1 pmol/L.

### Statistical Analysis.

No statistical method was used beforehand to determine sample size. The investigators were not blinded and no data points were excluded. Data are represented as mean ± SD. The number of duplicates as well as the type of test performed is indicated in each figure legend.

## Supplementary Material

Supplementary File

Supplementary File

Supplementary File

Supplementary File

## Data Availability

Bulk and single-cell mRNA-sequencing data are deposited at Gene Expression Omnibus (GEO) and are publicly available under SuperSeries GSE183964 (https://www.ncbi.nlm.nih.gov/geo/query/acc.cgi?acc=GSE183964). Separately, mouse bulk mRNA-sequencing data are available under GSE183962 (https://www.ncbi.nlm.nih.gov/geo/query/acc.cgi?acc=GSE183962), human bulk mRNA-sequencing data are available under GSE183961 (https://www.ncbi.nlm.nih.gov/geo/query/acc.cgi?acc=GSE183961), and single-cell mRNA-sequencing data are available under GSE183963 (https://www.ncbi.nlm.nih.gov/geo/query/acc.cgi?acc=GSE183963).

## Change History

January 21, 2022: The byline has been updated.
