# Supplementary material for: Adult mouse and human organoids derived from thyroid follicular cells and modeling of Graves’ hyperthyroidism
Source: Proc Natl Acad Sci U S A. 2021 Dec 16;118(51):e2117017118. doi: 10.1073/pnas.2117017118 (PMC8713972; doi:10.1073/pnas.2117017118)
Supplement: Supplementary File [file pnas.2117017118.sapp.pdf]

Supplementary Information for

Adult mouse and human organoids derived from thyroid follicular cells and modelling of Graves' hyperthyroidism

Jelte van der Vaart[1,2], Lynn Bosmans[1], Stijn F. Sijbesma[1], Kèvin Knoops[3], Williine J. van de Wetering[3], Henny G. Otten[4], Harry Begthel[1,2], Inne H.M. Borel Rinkes[5], Jeroen Korving[1,2], Eef G.W.M. Lentjes[6], Carmen Lopez-Iglesias[3], Peter J. Peters[3], Hanneke M. van Santen[7,8], Menno R. Vriens[5], Hans Clevers[1,2,7]*

[1] Hubrecht Institute, Royal Netherlands Academy of Arts and Sciences (KNAW) and University Medical Centre Utrecht, 3584 CT Utrecht, the Netherlands
[2] Oncode Institute, Hubrecht Institute, 3584 CT Utrecht, the Netherlands
[3] The Maastricht Multimodal Molecular Imaging Institute, Maastricht University, 6229 ER Maastricht, the Netherlands
[4] Central Diagnostic Laboratory (CDL) / Centre of Translational Immunology (CTI), UMCU, The Netherlands
[5] Department of Surgical Oncology, UMC Utrecht Cancer Center, Utrecht, The Netherlands
[6] Central Diagnostic Laboratory (CDL), University Medical Centre, Utrecht, The Netherlands
[7] Princess Máxima Center for Paediatric Oncology, Utrecht, The Netherlands
[8] Department of Paediatric Endocrinology, Wilhelmina Children Hospital, University Medical Center Utrecht, Utrecht, The Netherlands

*Corresponding author
Email: h.clevers@hubrecht.eu.

This PDF file includes:

Supplementary text
Legends for Datasets S1 to S3
Figures S1 to S5 including legends

Other supplementary materials for this manuscript include the following:

Datasets S1 to S3

Supplementary materials and methods

Thyroid organoid cultures

Digestion medium consisted of 1 mg/mL collagenase (Sigma-Aldrich, C9407) with 10 μM ROCK inhibitor Y-27632 (Abmole, M1817) and 100μg/mL Primocin (Invivogen, ant-pm-1) in AdDMEM/F12 (GIBCO)

Mouse expansion medium consisted of AdDMEM/F12 (GIBCO) supplemented with B27 supplement (Gibco, 17504044), Glutamax, HEPES, 100 U/mL Penicillin-Streptomycin (all Thermo Fisher scientific), 1.25 mM N-acetylcysteine (Sigma-Aldrich, A9165), 10 mM nicotinamide (Sigma-Aldrich, N0636) and the following growth factors: 1% R-spondin 3 conditioned medium (U-Protein Express), 0.5 µM A83-01 (Tocris, 2939), 1 µM FSK (Tocris, 1099), 500ng/mL EGF (Peprotech, AF-100-15-1mg) and 100 ng/mL FGF10 (Peprotech, 100-26).

Human expansion medium contained AdDMEM/F12 (GIBCO) supplemented with B27 supplement (Gibco, 17504044), Glutamax, HEPES, 100 U/mL Penicillin-Streptomycin (all Thermo Fisher scientific), 100 µg/mL Primocin (Invivogen), 1.25 mM N-acetylcysteine (Sigma-Aldrich, A9165), 10 mM nicotinamide (Sigma-Aldrich, N0636) and the following growth factors: 1% R-spondin 3 conditioned medium (U-Protein Express), 0.5 µM A83-01 (Tocris, 2939), 1 µM FSK (Tocris, 1099), 500ng/mL EGF (Preprotech, AF-100-15-1mg), 100 ng/mL FGF10 (Peprotech, 100-26), 3mM p38 inhibitor (Sigma-Aldrich, S7067), 1:1000 Wnt surrogate (U-Protein Express) and 100ng/mL TSH (Sigma-Aldrich, T8931).

21-40 days after seeding, human and mouse organoids were removed from the BME, mechanically dissociated into small fragments using a narrowed Pasteur pipette and re-seeded in fresh BME. Passage was performed in 1:3 – 1:5 split ratio once every 7 - 14 days for >1 year. During culturing, medium was refreshed at most every three days. Images of organoid cultures were taken on EVOS FL Cell Imaging System (Thermo Fisher scientific).

<u>Viability assays</u>

Cell viability was determined using CellTiterGlo 3D (Promega) according to the manufacturer's instructions. In short, organoids were seeded using the same protocol as mentioned before. Two hours after seeding, medium was removed from organoid plates and 200 µl CellTiterGlo 3D solution was added. Solution was incubated for 20min at RT while shaking (300rpm). 20 µl solution, without touching BME dome was transferred to black 96-well plate. Luminescence was acquired with Berthold XS3 LB 960 within 5 min.

<u>Immunohistochemistry and imaging</u>

Antibodies used for immunohistochemistry included PAX8 (Abcam, ab124445), NKX2.1 (Abcam, ab76013), anti-TG (Abcam, ab156008) and KI67 (eBiosciences, 14-5698-82). Antibodies used for immunofluorescence included anti-PAX8 (Abcam, ab124445), anti-NKX2.1 (Abcam, ab76013), anti-TG (Abcam, ab156008), anti-ZO-1 (Life Technologies, 40-2200), anti-KI67 (eBiosciences, 14-5698-82), Phalloidin–Alexa488 (Life Technologies, A12379) and DAPI (Invitrogen). Secondary antibodies included Alexa Fluor 568 goat anti-rabbit IgG (Life Technologies, A11036), Alexa Fluor 647 goat anti-rabbit IgG (Life Technologies, A21245) and Alexa Fluor 647 goat anti-mouse IgG (Life Technologies, A21236)

<u>qPCR analysis</u>

Primers used for qPCR analysis were designed using NCBI primer design.

Table S1. Primer list for RT-qPCR analysis

**Gene**	**Species**	**Forward**	**Reverse**
Pax8	Mouse	CAGCCTGCTGAGTTCTCCAT	CTGTCTCAGGCCAAGTCCTC
Nkx2.1	Mouse	CAGTTCCACTCTGCAACGGA	TGATTCGGCGTCGGCTGG
Tg	Mouse	TCCGGAGGAAAGTTGTGCTG	GCCGCTCACACTCAAAGAAC
Tpo	Mouse	TGGATAGTAATTTGCAGGTGGACA	CCTCTATTCGCACAGGAGGAC
Tshr	Mouse	ATGACACGGAAGTGGCTCTG	ACTCTGCACTTCTCTCTCCTTC
Calca	Mouse	AGGATCAAGAGTCACCGCTTC	CATGATGCCTTCCTGGTGAG
PAX8	Human	CATTTGAGCGGCAGCACTAC	AAGGGTGAGTGAGGATCTGC
NKX2.1	Human	CTCGCTCGCTCATTTGTTGG	TCGGCGGCGGCTGAG
TG	Human	GCCTGTGGCTTGTCTGTCA	GCGTAGTCCCCTGAATCCTG
TPO	Human	AGTGCAGTTGGCTGAGAAGA	TCCCTCTCGAGATGAAGGGG
TSHRa	Human	GCTTGCTGGACGTGTCTCAA	CCCGTGTGAGGTGAAGGAAA

SLC5A5	Human	ATGTCGGCCGTGCAGGT	CGCATCTCCAGGTACTCGTAGG
SLC26A4	Human	GCGAGCAGAGACAGGTCAT	CTCGTGCTGTTGCTGGAAAG
SLC16A2	Human	CCCCAAGCAAGAGAGGTGTC	CCTCCACATACTTCATCAGGTG

<u>Single-cell mRNA sequencing</u>
After sorting as indicated in material and methods, cells were first lysed for 5 minutes at 65 °C, and RT and second-strand mixes were dispensed by the Nanodrop II liquid handling platform (GC Biotech). Single-cell double-stranded cDNAs were pooled together and in vitro transcribed for linear amplification. Illumina sequencing libraries were prepared using the TruSeq small RNA primers (Illumina) and these DNA libraries were sequenced paired-end, respectively, on the Illumina NextSeq.

Fastq files were trimmed with TrimGalore-0.4.3 and mapped with STAR-2.5.3a to the mouse reference genome (GRCm38). Introns and exons were extracted from the corresponding annotation files. Cells with raw transcript counts higher than 1.5 times the interquartile range above the upper quartile of the rest of the population were removed as potential doublets. Subsequently, clustering and single-cell analysis were performed with Seurat package. To directly compare organoid and tissue cells in the violin plots, cells identified in the above-mentioned analysis were retrieved from another analysis containing both tissue and organoid cells ran with mintrans = 1500. Then, normalized expression (ndata) was plotted.

Legends for Datasets S1 to S3

Dataset S1. Normalised reads of mouse thyroid tissue and organoids bulk mRNA sequencing

Dataset S2. Normalised reads of human thyroid tissue and organoids bulk mRNA sequencing

Dataset S3. Differential expression analysis of cluster 5 compared to all other cluster in mouse TFC single cell mRNA sequencing

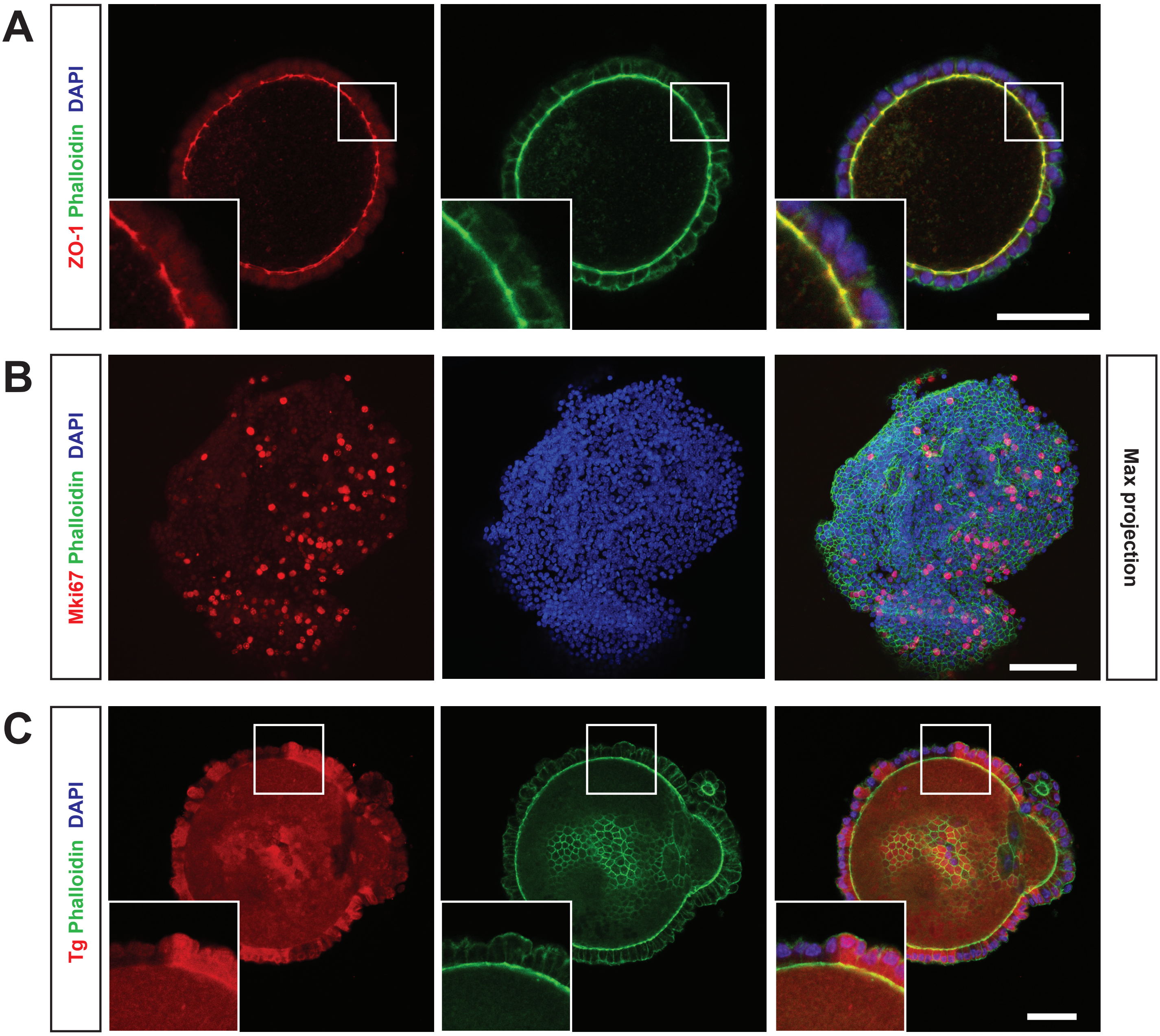

Fig. S1. TFCOs are comprised of polarised TFCs subdivided into proliferative and Tg-expressing TFCs.

A) Mouse TFCOs have apical-in morphology based on apical membrane marker ZO-1 (red). Overlap of ZO-1 and membrane stain Phalloidin (green) is only observed on inner surface of TFCOs. DAPI = nuclear stain (blue). Representative image of mouse TFCOs in passage 5 (mThy15). Scale bar = 50um

B) Proliferative cells are observed in mouse TFCOs as indicated by Mki67-positivity (red). Only a subpopulation of TFCs are proliferative per TFCO. Representative image of mouse TFCOs in passage 8 (mThy14). Image shows max projection of z-stack. Phalloidin = membrane stain (green), DAPI = nuclear stain (blue). Scale bar = 100um.

C) Subpopulations of mature TFCs can be identified in TFCOs in early days after organoid formation (day 5 after passaging) based on Tg. Representative image of mouse TFCOs in passage 5 (mThy16). Phalloidin = membrane stain (green), DAPI = nuclear stain (blue). Scale bar = 50um.

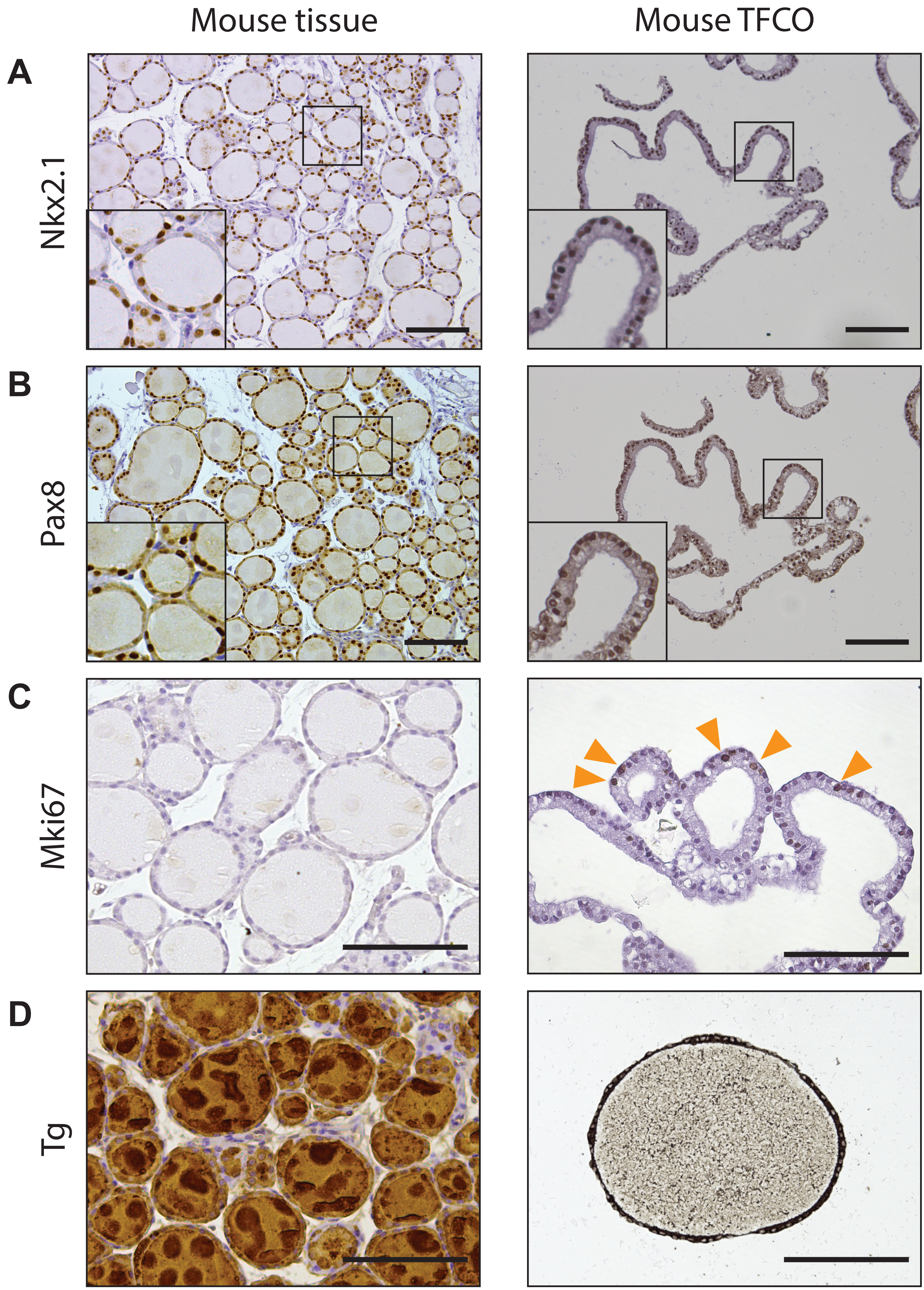

Fig S2. Mouse TFCs express nuclear thyroid transcription factors in both tissue and TFCOs while proliferation is limited to TFCOs.
A-D) Representative images of mouse primary thyroid tissue and TFCOs stained for thyroid transcription factors Nkx2.1 (A), Pax8 (B), Mki67 (C) and Tg (D). Both transcription factors show nuclear expression in both mouse tissue as mouse TFCOs. Proliferation marker Mki67 is only observed in some cells in TFCOs and not observed in mouse primary thyroid tissue. Tg expression is observed in colloid follicles in tissue samples and similary in the lumen of TFCOs. Scale bar = 100um.

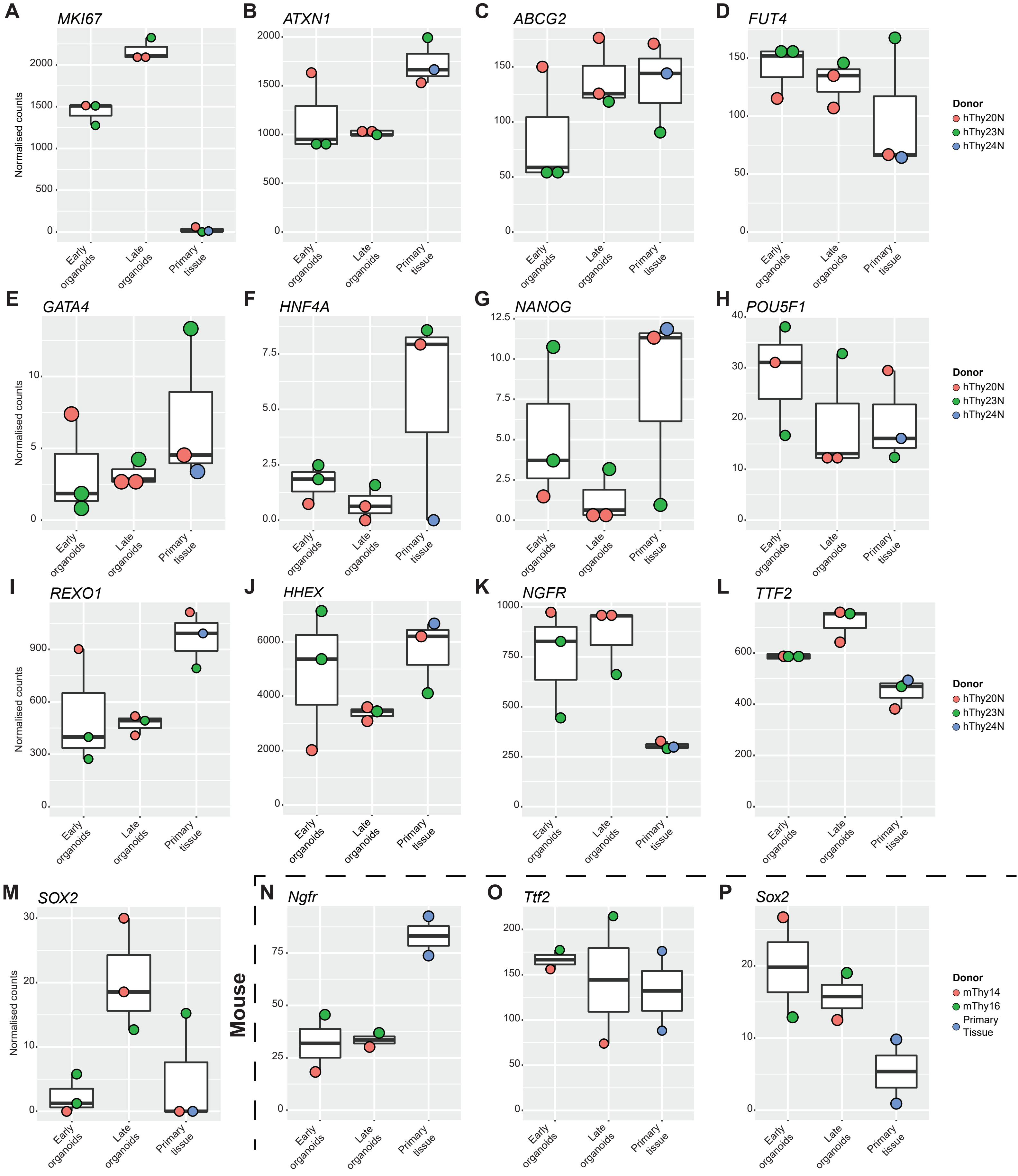

Fig S3. Most proposed stem cell markers show no enrichment in human TFCOs in early and late passages except for NGFR, TTF2 and SOX2 which are not enriched in mouse TFCOs

A-P) Boxplots of human (A-M) and mouse (N-P) primary thyroid tissue (Primary tissue, early passage TFCOs (Early organoids) and late passage TFCOs (Late organoids) normalised counts of bulk mRNA sequencing. Colour of the dots indicates donor visualised as described on the right of the row of graphs. Boxplot shows median, two hinges (25th and 75th percentile) and two whiskers (largest and smallest value no further than 1.5x inter-quartile range).

A) Increased proliferation is observed over time from primary thyroid tissue to early and late organoids by normalised counts of *MKI67* in line with observed increase in TFCO growth over passages.

B-I) Proposed stem cell markers *ATXN1* (B), *ABCG2* (C), *FUT4* (D), *GATA4* (E), *HNF4A* (F), *NANOG* (G), *POU5F1* (H), *REXO1* (I) and *HHEX* (J) are not enriched in correlation with *MKI67* and time in human tissue and TFCOs.

K-M) Proposed stem cell markers *NGFR* (K) and *TTF2* (L) are enriched in TFCOs compared to human tissue and increased over time and SOX2 (M) is enriched in late passage TFCOs. *NGFR, TTF2* and *SOX2* expression is therefore correlated to increased proliferation.

N-P) Potential human stem cell markers *Ngfr* (N), *Ttf2* (O) and *Sox2* (P) are not enriched in mouse TFCOs compared to primary tissue and increased over time in culture.

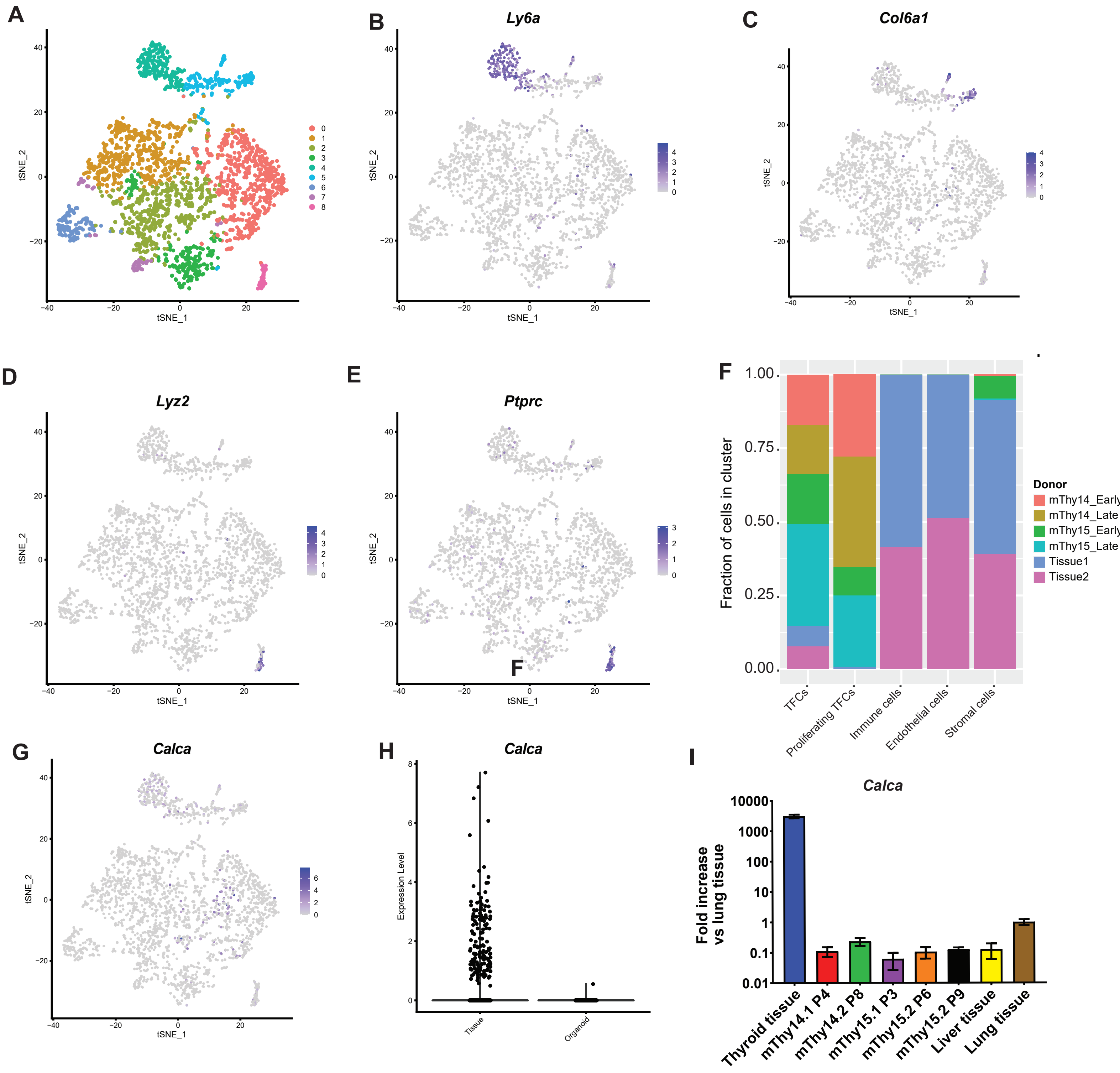

Fig S4. Single cell sequencing of mouse primary tissue and TFCOs

A) tSNE representation of 9 clusters of mouse thyroid tissue cells (n = 747) and mouse TFCO cells (n=1843).

B) tSNE representation of the expression of the endothelial cell marker *Ly6a* (linear scale)

C) tSNE representation of the expression of the stromal cell marker *Col3a1* (linear scale)

D) tSNE representation of the expression of the immune cell marker *Lyz2* (linear scale)

E) tSNE representation of the expression of the immune cell marker *Ptprc* (linear scale)

F) Clusters marked as endothelial, immune and stromal cells after single cell mRNA sequencing are comprised of cells originating from both primary tissues but not from TFCOs. The TFC cluster contains cells from all samples: primary tissue, early passage TFCOs and late passage TFCOs. Proliferative TFCs are only identified in cells from early and late passage TFCOs. Colour indicates donor.

G) tSNE representation of the expression of the C-cell hormone calcitonin *Calca* (linear scale).

H) Violin plots of the normalized expression level of *Calca* indicates potential presence of C-cells in tissue but absence in TFCOs.

I) RT-qPCR analysis reveals loss of *Calca* expression in mouse TFCOs compared to primary thyroid tissue in early (<4 passages) and late (>6 passages) TFCOs. Expression levels are relative to lung tissue expression levels. N=3. Error bars = stdev.

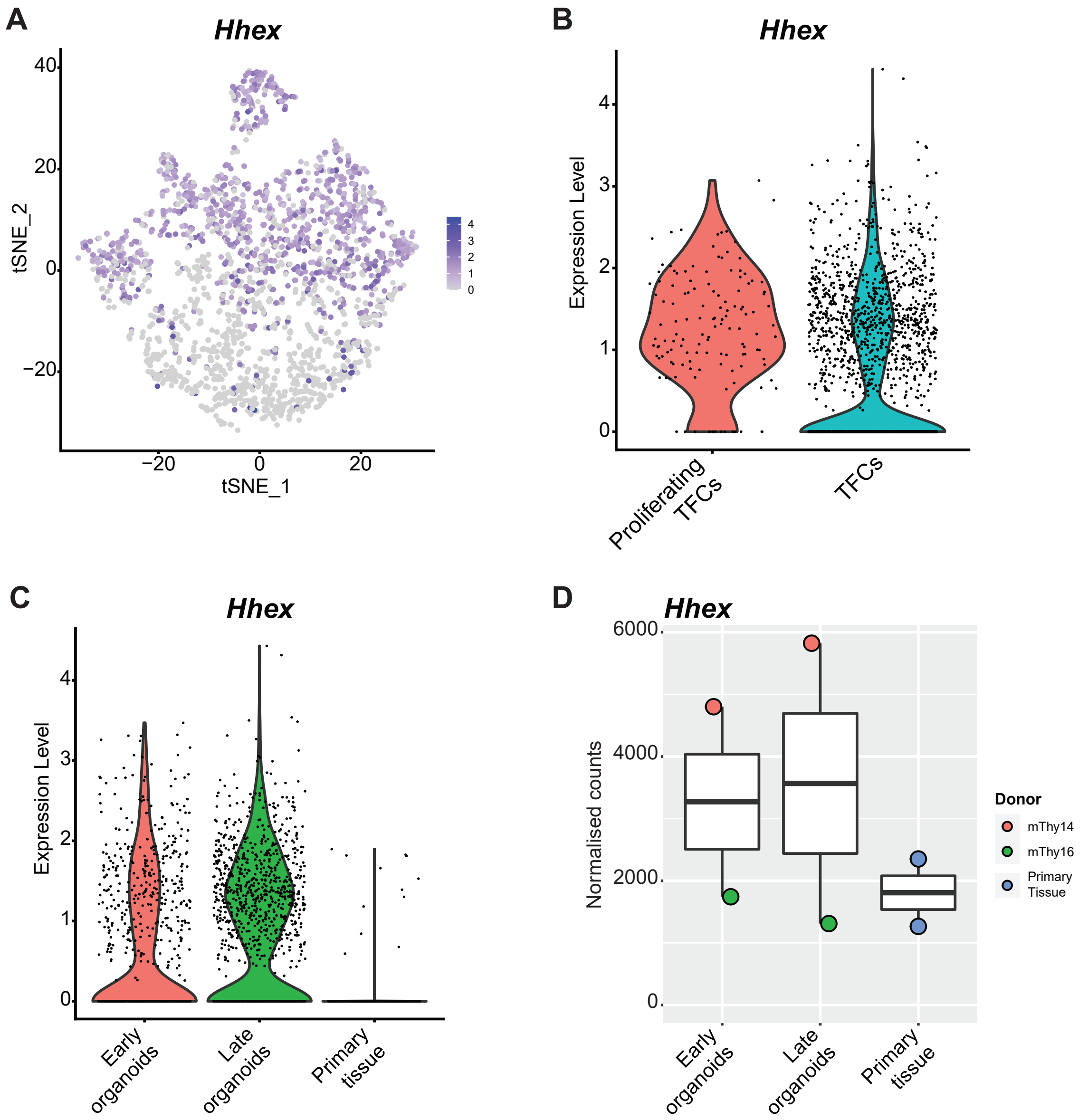

Fig S5. Single cell sequencing of mouse primary tissue and TFCOs reveals Hhex as potential mouse thyroid stem cell marker

A) tSNE representation of the expression of the potential stem cell markers *Hhex* enriched in proliferative TFCs (linear scale).

B) Violin plots of the normalized expression level of *Hhex* in proliferative TFCs (n = 136 cells) and TFCs (n = 1,983 cells) shows increased expression in proliferative TFCs compared to other TFCs.

C) Violin plots of the normalized expression level of *Hhex* in early passage TFCOs ("Early organoids", n = 725 cells), late passage TFCOs ("Late organoids", n= 1101 cells) and primary thyroid tissue ("Primary tissue", n = 293 cells) show presence of Hhex expression only in TFCOs and increased expression over time.

D) Boxplot of *Hhex* expression in mouse primary thyroid tissue and early, and late passage TFCOs using bulk mRNA sequencing. A trend can be observed of increased expression from tissue to TFCOs and increased expression over time in TFCOs. Boxplot shows median, two hinges (25th and 75th percentile) and two whiskers (largest and smallest value no further than 1.5x inter-quartile range).
